# A Smartphone-Delivered Program (Anathema) to Promote the Sexual Health of Older Adults, Colorectal Cancer Survivors, and Stroke Survivors: Protocol for a Feasibility Pilot Randomized Controlled Trial

**DOI:** 10.2196/46734

**Published:** 2023-06-27

**Authors:** Cristina Mendes-Santos, Ana Luísa Quinta-Gomes, Raquel Pereira, Priscila Vasconcelos, Pedro Nobre, Joana Couto, Ana Correia de Barros

**Affiliations:** 1 Fraunhofer Portugal Center for Assistive Information and Communication Solutions Porto Portugal; 2 Center for Psychology at the University of Porto Porto Portugal

**Keywords:** colorectal cancer, internet interventions, smartphone, older adults, participatory design, randomized controlled trial, stroke, survivors

## Abstract

**Background:**

Despite the prevalence of sexual distress and dysfunction in older adults in general and stroke and colorectal cancer survivors in particular, access to specialized care is limited by organizational barriers and stigma, embarrassment, and discrimination. The internet allows reaching services that would otherwise be difficult or impossible to reach, and as smartphones are personal (intimate) technologies, they are a promising vehicle to close this gap. However, research focusing on smartphone-delivered sexual health promotion programs is scarce.

**Objective:**

This study aims to assess the acceptability, feasibility, and preliminary efficacy of Anathema, an 8-week, iOS/Android smartphone–delivered, individually tailored, cognitive-behavioral sexual health promotion program developed to improve relationship and sexual satisfaction, sexual functioning, sexual distress, sexual pleasure, and health-related quality of life (HRQoL) in older adults, colorectal cancer survivors, and stroke survivors compared to treatment as usual in a waiting-list control condition.

**Methods:**

Two-arm, parallel, open-label, waiting list, feasibility, pilot randomized controlled trials (RCTs) will be conducted involving older adults, stroke survivors, and colorectal cancer survivors. The primary outcomes are the acceptability, usability, and feasibility of Anathema. Sexual function, relationship and sexual satisfaction, sexual pleasure, sexual distress, anxiety, depression, and HRQoL are the secondary outcomes. This study has been reviewed and approved by the ethics committees of Instituto Português de Oncologia do Porto Francisco Gentil, Europacolon Portugal, Faculty of Psychology and Educational Sciences, University of Porto, and Sigmund Freud University (approval numbers: CES218R/021, CES19/023, and 2022/01-05b).

**Results:**

This project is funded by the European Commission through the Active and Assisted Living (AAL) Programme (reference: AAL-2020-7-133-CP) from April 2021 to December 2023. Recruitment for the pilot RCTs started on January 2023 in Portugal, Austria, and the Netherlands and is currently ongoing. As of May 2023, we randomized 49 participants in the trials. We expect to complete the RCTs in September 2023. The results on the acceptability, feasibility, and preliminary efficacy of Anathema are expected in the second semester of 2023. We expect Anathema to be highly accepted by the populations under study; to prove feasible to scale up to parent RCTs; and to be potentially efficacious in improving sexual functioning, relationship and sexual satisfaction, sexual distress, sexual pleasure, and HRQoL in older adults, colorectal cancer survivors, and stroke survivors compared to treatment as usual in a waiting-list control condition. The study results will be published in open-access venues according to COREQ (Consolidated Criteria for Reporting Qualitative Research) and CONSORT EHEALTH (Consolidated Standards of Reporting Trials of Electronic and Mobile Health Applications and Online Telehealth) guidelines.

**Conclusions:**

The study results will inform the refinement and scale-up of Anathema. Anathema’s wider-scale implementation can potentially promote the sexual health of largely neglected user groups such as older adults, colorectal cancer survivors, and stroke survivors.

**International Registered Report Identifier (IRRID):**

DERR1-10.2196/46734

## Introduction

### Background

Sexual health is an integral component of general health [1]. It is significantly related to physical and psychological health, as well as to the perception of well-being and health-related quality of life (HRQoL) [1-3]. Being sexually active has been reported to be associated with improved mental health, increased heart rate variability, and lower risk of certain cancers and coronary events, resulting in lower annual death rates [4]. However, the aging process frequently threatens the experience of a positive, healthy, and fulfilling sex life [5].

Due to physiological and psychosocial changes, older adults are at higher risk of developing sexual difficulties when compared to the general population [6,7]. Frequent problems affecting older women include decreased libido, lack of vaginal lubrication, and orgasmic difficulties. Among older men, erection problems, reduced sexual desire, and an inability to reach climax are the most reported sexual difficulties [5-7]. Previous studies focusing on the predictors of such conditions in both sex groups [5] have identified interpersonal and attitudinal factors, mental health conditions (eg, depression), and physical health conditions as being strongly associated with sexual problems. In this regard, colorectal cancer [8] and stroke [9] are examples of prevalent acquired medical conditions highly associated with older age, which may result in significant impairments in sexual functioning and satisfaction [10,11].

Studies targeting colorectal cancer survivors [12,13] often reported a decrease in the frequency of sexual intercourse, a high incidence of sexual problems (ie, low sexual desire, and arousal and orgasm difficulties), and a significant decrease in survivors’ sexual satisfaction after cancer treatment. Identified risk factors for sexual dysfunction include being diagnosed with low rectal cancers, undergoing abdominoperineal resection, having a stoma, and prior treatment with radiotherapy [13]. Moreover, male sex, older age, individual and partner distress, and comorbidities have been reported to be associated with increased sexual disorders in colon cancer survivors [12]. The literature also suggests that sexual difficulties in colorectal cancer survivors span the continuum of care [14].

Among stroke survivors, research reports a high prevalence of sexual dysfunction [15]. The etiology of poststroke sexual dysfunction is multifactorial, and it includes organic (eg, lesion site, comorbidities, medication, etc) and psychosocial factors (eg, anxiety, depression, fear of recurrence, etc) [15]. Common clinical presentations of sexual dysfunction in female survivors include decreased libido, lack of vaginal lubrication, arousal problems, and orgasmic dysfunction [11,15,16]. Male survivors often report decreased libido, sexual activity, and coitus frequency, as well as erectile and ejaculatory dysfunctions [11,15,16]. These difficulties also manifest in stroke survivors with an absent or mild physical impairment, with nearly half experiencing decreased libido, coitus frequency, sexual arousal, orgasm, and sexual satisfaction [11,16]. This finding suggests that changes to prestroke relationships and roles, communication difficulties between partners, taboo and stigma, negative beliefs and attitudes toward sexuality, and perceived social norms may disrupt sexual expression in stroke survivors, leading to decreased sexual satisfaction and poor HRQoL outcomes [11,17].

Despite the prevalence of sexual distress and dysfunction in older adults in general and stroke and colorectal cancer survivors in particular, access to specialized care is limited, and sexual health remains a largely neglected component of treatment [18]. Recent reviews [19-21] have identified a lack of available information concerning sexual issues, a lack of education and training of health care professionals on sexual health, poor quality of the relationship between patients and health professionals, and misalignment between their perspectives on the importance of sexual health as important barriers to accessing sexual health support. Negative societal views and beliefs toward sexual health, stigma, embarrassment, and discrimination were also identified as major factors hindering access to sexual health support and treatment among older adults and other chronic disease groups [19-21]. Finding alternative ways of facilitating access to evidence-based sexual health support that is tailored to the unmet needs of older adults, colorectal cancer survivors, and stroke survivors in a nonstigmatizing manner is, therefore, critical to overcome barriers to treatment-seeking and promote their sexual health.

Because smartphones are personal (intimate) technologies that, augmented with internet connections, allow reaching services that would otherwise be difficult or impossible to reach, they are a promising vehicle to close this gap. However, research focusing on internet-delivered interventions for sexual health is scarce [22]. Most studies addressing such programs focused on male and female sexual dysfunctions in adult populations [23], and only a few targeted sexual difficulties related to chronic conditions. A recent systematic review [24] of mobile health interventions for sexual health among adults with chronic diseases reported no studies with interventions delivered through smartphones. All analyzed studies used a website for intervention delivery, failing to leverage the advantages of mobile devices in this context, namely, “convenience, privacy, anonymity, and more interactive treatment for sexual dysfunction” [24]. Still, the evidence suggests that internet-delivered sex therapy is effective in improving psychosexual outcomes in chronic patients [24].

Among these, cancer is the most frequently researched condition [25-27], with studies including patients with gynecological [28,29], breast [29-32], prostate [33], and colorectal cancers [28]. The findings of these studies suggest that internet-delivered interventions for sexual health are feasible, acceptable, and potentially effective in improving sexual health in cancer survivors [25,27]. Reviewed studies have reported improvements in sexual function and activity [28,29,31]; sexual arousal and pleasure [30]; sexual, intimacy, and relationship satisfaction [34,35]; and sexual distress [28,30]. Furthermore, the positive effects of these interventions in cancer survivors are likely to persist in the long term [34].

However, previously conducted studies vary greatly in sample size, intensity, and program length, and further research is necessary to attest the feasibility and efficacy of internet-delivered interventions for sexual health among different target cancer populations, including colorectal cancer populations, and the use of digital technologies like smartphones.

To the best of our knowledge, no study has yet assessed the efficacy of internet-delivered interventions to promote sexual health in stroke survivors or older adults. Nevertheless, professionally guided online sexual rehabilitation programs were identified as highly consensual in a recent Delphi study involving stroke survivors, their partners, clinicians, and researchers [36], underlining the pertinence of developing and testing internet-delivered sexual health promotion programs targeting stroke survivors.

To address the research and supportive care gaps mentioned above, we developed, in the context of a European project named *Anathema – Technology for ageless sexual health*, an 8-week, iOS/Android smartphone–delivered, individually tailored, cognitive-behavioral, sexual health promotion program to improve relationship and sexual satisfaction, sexual function, sexual distress, sexual pleasure, and HRQoL in older adults, colorectal cancer survivors, and stroke survivors (for further details on Anathema’s development and structure, please see [37]). This project is funded by the European Commission through the Active and Assisted Living (AAL) Programme. This report presents the research protocol that will support the assessment of Anathema’s acceptability, feasibility, and preliminary efficacy in the 3 target populations compared to treatment as usual (TAU) in a waiting-list control (WLC) condition. The findings of this study will support Anathema’s future scale-up, implementation, and commercialization, and ultimately facilitate access of older adults, colorectal cancer survivors, and stroke survivors to sexual health support.

### Objectives and Hypothesis

This study aims to assess the acceptability, usability, feasibility, and preliminary efficacy of Anathema in older adults, colorectal cancer survivors, and stroke survivors compared to TAU in a WLC condition.

We hypothesize that Anathema will be highly accepted by study participants and prove feasible to scale up. We also expect Anathema to preliminarily show more efficacy in improving relationship and sexual satisfaction, sexual functioning, sexual distress, sexual pleasure, and HRQoL in the study populations compared to TAU in a WLC condition.

## Methods

### Study Design

**Figure 1:**
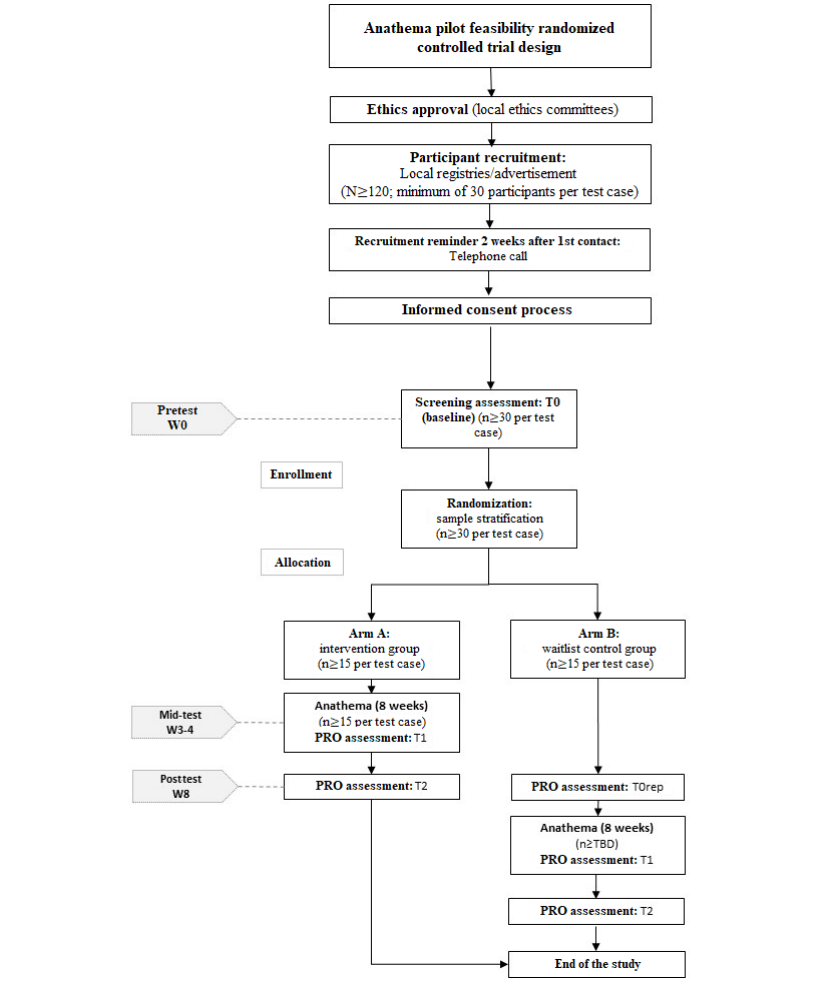
Anathema study design. PRO: participant-reported outcome; TBD: to be determined.

Two-arm, parallel, open-label, waiting list, feasibility, pilot randomized controlled trials (RCTs) (Figure 1) will be conducted with older adults, stroke survivors, and colorectal cancer survivors. We will have 1 test case per target population, where different service models (unguided, guided, or blended care) will be tested. The feasibility pilot trials will be performed to identify weaknesses in the study design, test the data collection process, estimate sample sizes, and assess the feasibility of the parent RCTs. The acceptability of Anathema by study participants; the structure, format (ie, unguided, guided, and blended), and content of the study intervention; and between-group differences will also be preliminarily evaluated through this design.

### Ethics Approval

This study has been reviewed and approved by the ethics committees of Instituto Português de Oncologia do Porto Francisco Gentil (IPO-Porto) (approval number: CES218R/021, and CES19/023), Europacolon Portugal, Faculty of Psychology and Educational Sciences, University of Porto (approval number: 2022/01-05b), and Sigmund Freud University.

### Study Population and Research Sites

This research will target 3 study populations: older adults, colorectal cancer survivors, and stroke survivors. Older adults will be recruited in the Netherlands and Austria. Colorectal cancer survivors and stroke survivors will be recruited in Portugal. A minimum of 30 participants [38,39] per target group is expected to participate in the study trials.

The criteria presented in Textbox 1 will be used to check eligibility for participation in the study:

The study will be conducted at 5 different research sites: Unie KBO in the Netherlands; Instahelp in Austria; and IPO-Porto, Europacolon Portugal, and Centro de Reabilitação Profissional de Gaia in Portugal. Sites invited to participate were selected owing to their working standards and resources.

**Eligibility criteria for Anathema’s feasibility randomized controlled trials.**

**Inclusion criteria**

- All target groups: Ability to provide informed consent; age ≥55 years; all gender identities, sexual orientations, relationship statuses, and sexual practices at the time of the study; absence of severe psychiatric comorbidity (psychotic or bipolar disorders, severe depression, and substance abuse); single or partnered; no unstable medical conditions precluding participation in a sexual health promotion program; no concurrent therapy to alleviate problems with sexuality or intimacy, or cognitive-behavioral therapy for other psychological issues; ongoing regular psychoactive medication accepted if the dosage has been stable during the last 3 months; daily access to the internet and ability to perform basic use of a smartphone; and no concurrent participation in any other interventional study or clinical trial.
- Specific for colorectal cancer survivors: History of histologically confirmed colorectal cancer; an interval of ≥6 months from primary adjuvant treatment completion (surgery, chemotherapy, or radiotherapy); no previous treatment for another type of cancer (except for cervix carcinoma in situ or basal cell carcinoma); and disease-free at the time of study entry.
- Specific for stroke survivors: Confirmed diagnosis of stroke (hemorrhagic or ischemic) based on clinical examination and imaging, as assessed by a neurologist; and absence of severe cognitive issues or dementia.

**Exclusion criteria**

- All target groups: Age <55 years; inability to co-operate and provide informed consent; current severe, uncontrolled systemic disease or mental disorder; parallel ongoing psychological treatment; ongoing regular psychoactive medication if the dosage has been changed during the last 3 months; no access to the internet; inability to use a smartphone and the internet; parallel ongoing participation in another interventional study or clinical trial; and assessment by the investigator to be unable or unwilling to comply with the requirements of the protocol.
- Specific for colorectal cancer survivors: Colorectal cancer not histologically or cytologically confirmed; history of other malignancy within the last 5 years; and metastasized colorectal cancer.
- Specific for stroke survivors: Unconfirmed stroke diagnosis; and severe cognitive impairment or dementia.

### Study Intervention and TAU

**Figure 2:**
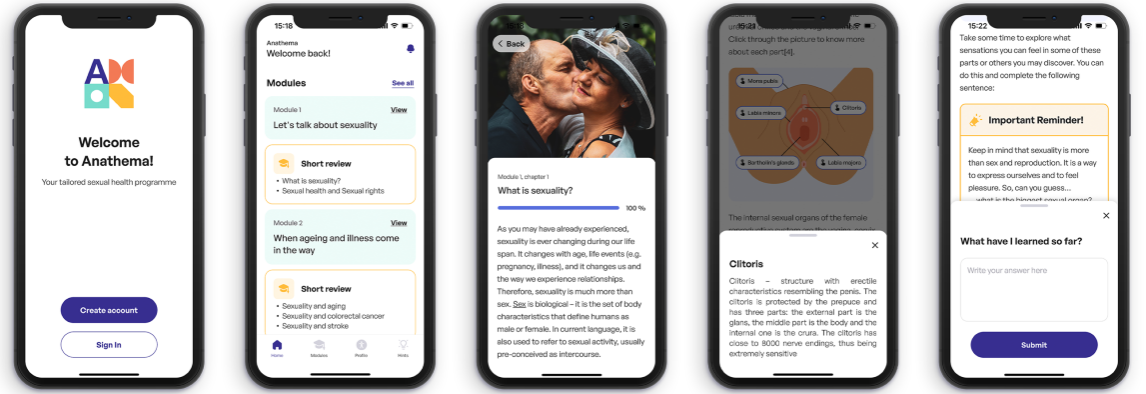
Anathema app overview.

Anathema (Figure 2) is an 8-week, iOS/Android smartphone–delivered, individually tailored, cognitive-behavioral sexual health promotion program developed to improve relationship and sexual satisfaction, sexual functioning, sexual distress, sexual pleasure, and HRQoL in older adults, colorectal cancer survivors, and stroke survivors.

The program was created using a participatory design approach [40], involving its primary (older adults, colorectal cancer survivors, and stroke survivors), secondary (psychologists and sexologists), and tertiary end users (managers of care homes, day-care centers, or clinics). Anathema’s development followed an agile development framework [41] and was based on peer-review sources (eg, [12,42-49]) and user research insights (eg, [37]). The program adopts a transdiagnostic structure, featuring sexual health promotion strategies considered appropriate for the different populations under study. Its central components are psychoeducation, cognitive restructuring, mindfulness, communication skills training, exercises to promote sexual sensation awareness and erotic repertoire, and masturbatory and sensate focus exercises. Anathema is structured into 5 modules to be completed for up to 8 weeks. A detailed description of Anathema’s structure and content is provided in Table 1.

Regarding human support, Anathema may be delivered using 3 service models. The subtypes are as follows: unguided or self-guided (the primary end user follows all the intervention programs on her/his own with no support from a clinical psychologist), guided (the intervention program incorporates clinical psychologists’ synchronous or asynchronous support, guidance, and feedback via email, chat, etc), and blended (treatment programs that combine face-to-face or videoconference sessions with online treatment modules in an integrated and sequential format). An iOS/Android app co-developed in the context of this project will be used to deliver the self-guided version of the intervention to older adults in the Netherlands. In this approach, no clinical psychologist-participant contact is foreseen. This app will also guide colorectal cancer and stroke survivors throughout the program in Portugal. Synchronous (eg, voice and chat) and asynchronous (eg, email) communication channels will be available to provide participants with guidance and support weekly and upon request. The blended-care format intervention will be delivered to older adults in Austria via an iFrame to be made available on the Instahelp platform [50]. In this case, the developed modules will be combined with 3 videoconference sessions (at the onset, mid, and conclusion of the intervention) focusing on the clarification of the intervention’s content and strategies to be implemented. Upon request, sessions are also possible for participants requiring additional support from clinical psychologists.

In this research, TAU refers to the routine sexual health care participants would receive at the recruitment sites. Therefore, the type of TAU received may vary and include pharmacologic treatment or psychosocial support. WLC participants will be informed that they are free to access any form of support during the study and encouraged to seek professional help in case they experience symptomatic deterioration or other difficulties. WLC participants will be offered the same treatment protocol applied to the experimental group after completion of the program (approximately 8 weeks from baseline).

**Table 1:** Anathema’s structure and content.

Module	Intervention components	Content
Let’s talk about sexuality? (week 1)	Psychoeducation	Concepts underlying sexuality Male and female anatomies and erogenous zones Sexual response and sexual pleasure Sexual health, sexual rights, and their importance Activities: Setting my personal goals within Anathema, and I get pleasure from…
When aging and illness come in the way (week 2)	Psychoeducation (tailored to each study population)	Successful aging Sexual expression in older age Physiological, cognitive, and emotional changes in older age Sexual problems and sexual dysfunction in older age Biopsychosocial changes associated with colorectal cancer (CRC) and stroke, and their impact on survivors’ sexual health Activities: Practicing self-compassion, and identifying and classifying the changes I experience in my sexual life due to aging, CRC, or stroke
Emotional and physical intimacy (weeks 3-6)	Psychoeducation Cognitive restructuring Mindfulness Communication skills training Sensate focus	Sexual beliefs, thoughts, and emotions, and their impact on sexuality Identifying, evaluating, and modifying maladaptive thinking Interpersonal relationship and communication issues commonly associated with aging, CRC, and stroke Restoring interpersonal relationships and intimacy Activities: Rational disputation, diary of thoughts and emotions, practicing mindfulness, identifying and overcoming relationship stressors, improving communication, and sensate focus
Exploring one’s sexuality (week 7)	Psychoeducation Mindfulness exercises Sexual skills training	Giving and receiving pleasure: eroticism, masturbation, and intercourse Sex aids and strategies to enhance sexual pleasure and satisfaction Activities: Practicing mindfulness and identifying treatment options
Planning for a long-term fulfilling sex life (week 8)	Relapse prevention	Overview of Anathema and progress evaluation Strategies to maintain progress and prevent setbacks Promotion of a healthy lifestyle as key to better sexual health Activities: Managing therapeutic progress, and strategies to enhance sexual pleasure and satisfaction

### Measures

#### Primary Outcome Measures

The primary outcomes in this study are the acceptability, usability, and feasibility of Anathema. To assess Anathema’s acceptability, participants will self-report on the appropriateness and usefulness of the intervention’s content, length, usability, and service delivery model, using a self-developed 5-point Likert scale and the System Usability Scale [51].

Anathema’s feasibility-specific measures include recruitment, randomization, retention, and adherence rates (Table 2). Postintervention semistructured interviews will also be conducted to gather further data on Anathema’s usefulness, usability, and feasibility, as perceived by participants.

**Table 2:** Primary outcome measures.

Outcome	Metrics
Acceptability	Self-developed 5-point Likert scale assessing the appropriateness and usefulness of the intervention’s content, length, usability, and service delivery model Postintervention semistructured interviews
Usability	System Usability Scale [51]
Feasibility	Recruitment: Number of participants screened per week Number of screening failures Number of participants enrolled per week Proportion of recruited participants opting to enroll Randomization: Proportion of eligible participants who access Anathema following randomization ‎Proportion of eligible participants who complete at least one module of Anathema Retention: Proportion of participants who complete T0 (baseline) measures Condition-specific completion rates following randomization (completion is defined as ≥50% of modules accessed at least once) ‎Proportion of participants initiating baseline measures who complete T1 (midtest) and T2 (posttest) measures Adherence: Proportion of participant dropouts from each intervention condition Proportion of participant withdrawals from the study Postintervention semistructured interviews

#### Secondary Outcome Measures

Sexual function, relationship and sexual satisfaction, sexual pleasure, sexual distress, anxiety, depression, and HRQoL are secondary outcome variables in this research. A summary of the secondary outcome measures to be used in this study is presented in Table 3. Participants will not be invited to fill in all the listed measures, since secondary outcome measures will be tailored to participants’ gender, sexual orientation, and condition.

**Table 3:** Secondary outcome measures.

Measure	Construct	Number of items	Dimensions	Rating scale	Cutoff points	Psychometric properties	Translation/adaptation to the target populations
Sociodemographic and clinical background questionnaires tailored to each target group	N/A^a^	11	N/A	N/A	N/A	N/A	Original version developed in Portuguese (Portugal) by the research team and translated to Dutch and German.
Mini-International Neuropsychiatric Interview (MINI) [52]	Mental disorders	16 modules (branching tree logic items)	Multidimensional	Dichotomous rating scale	N/A	Cohen k=0.7	Translated and adapted to Portuguese (Portugal), Dutch, and German.
Patient Health Questionnaire (PHQ-9) [53]	Depression	9	Unidimensional	4-point Likert scale	Mild depression (<5), moderate depression (6-10), moderately severe depression (11-15), and severe depression (>15)	α=.86	Translated and adapted to Portuguese (Portugal), Dutch, and German.
Generalized Anxiety Disorder Screener (GAD-7) [54]	Anxiety	7	Unidimensional	4-point Likert scale	Normal anxiety (<5), mild anxiety (5-9), moderate anxiety (10-15), and severe anxiety (15-21)	Primary care setting (α=.92); general population (α=.89)	Translated and adapted to Portuguese (Portugal), Dutch, and German.
World Health Organization Quality of Life: Brief Version (WHOQoL-BREF) [55]	Quality of life	26	Multidimensional	5-point Likert scale	N/A	.66<α<.84	Translated and adapted to Portuguese (Portugal), Dutch, and German.
Global Measure of Sexual Satisfaction (GMSEX) [56]	Sexual satisfaction	5	Unidimensional	7-point Likert scale	N/A	α=.94	To be translated and adapted to Dutch and German in the context of this research.
Global Measure of Relationship Satisfaction (GMREL) [56]	Relationship satisfaction	5	Unidimensional	7-point Likert scale	N/A	α=.95	To be translated and adapted to Dutch and German in the context of this research.
Sexual Pleasure Scale (SPS) [57]	Sexual pleasure	3	Unidimensional	7-point Likert scale	N/A	α=.84	To be translated and adapted to Dutch and German in the context of this research.
International Index of Erectile Function (IIEF) [58]	Sexual function	15	Multidimensional	0-5 or 1-5 numeric rating scale	≤25 expresses risk of sexual dysfunction	α>.70 for all domains	Translated and adapted to Portuguese (Portugal), Dutch, and German.
Female Sexual Function Index (FSFI) [59]	Sexual function	19	Multidimensional	0-5 or 1-5 numeric rating scale	≤26 expresses risk of sexual dysfunction	α>.9 for all domains	Translated and adapted to Portuguese (Portugal), Dutch, and German.
Sexual Distress Scale (SDS-R) [60]	Sexual distress	13	Unidimensional	5-point Likert scale	N/A	α>.85	Translated and adapted to Portuguese (Portugal), Dutch, and German.

^a^N/A: not applicable.

### Study Procedures

#### Compliance With Laws, Regulations, and Ethical Standards

The study will comply with the following laws, regulations, and ethical standards: the International Conference on Harmonisation (ICH) E6 Guideline for Good Clinical Practice (GCP), the Declaration of Helsinki (October 1996), Regulation (EU) 2016/679 of the European Parliament and of the Council of 27 April 2016 on the protection of natural persons with regard to the processing of personal data and on the free movement of such data, and repealing Directive 95/46/EC (General Data Protection Regulation), as well as the following Portuguese Decree-laws: Lei n.º 58/2019 (Assegura a execução, na ordem jurídica nacional, do Regulamento (UE) 2016/679 do Parlamento e do Conselho, de 27 de abril de 2016, relativo à proteção das pessoas singulares no que diz respeito ao tratamento de dados pessoais e à livre circulação desses dados); Lei n.º 21/2014 (Lei da Investigação Clínica), de 16 de Abril; Lei n.º 73/2015, de 27 de julho (Primeira alteração à Lei n.º 21/2014, de 16 de abril, que aprova a lei da investigação clínica, no sentido de fixar as condições em que os monitores, auditores e inspetores podem aceder ao registo dos participantes em estudos clínicos); Regulamento n.º 258/2011 (nos termos do artigo 77.º do Estatuto da Ordem dos Psicólogos Portugueses, aprovado pela Lei n.º 57/2008, de 4 de Setembro, a Ordem elabora, mantém e atualiza o Código Deontológico da Ordem dos Psicólogos Portugueses); and Linhas de Orientação para a Prestação de Serviços de Psicologia Mediados por Tecnologias da Informação e da Comunicação (TIC) da Ordem dos Psicólogos Portugueses.

In Austria, clinical psychologists will follow the Ethics Guideline from the Federal Ministry of Labour, Social Affairs, Health, and Consumer Protection. The practice of the profession of clinical psychology and health psychology in Austria is regulated by the Psychologists Act 2013, Federal Law Gazette I No. 182/2013. The Code of Professional Conduct, in the form of an ethics guideline, supplements and concretizes the professional duties laid down in the Psychologists Act 2013 and serves to safeguard and promote the professional ethics of the members of the profession. The professional and ethical principles are based on the Meta Code of Ethics of the European Federation of Psychologists’ Associations (EFPA) as follows: (1) respect for the dignity and rights of the person, (2) competence, (3) responsibility, and (4) integrity. The most recent version (as of May 21, 2021) was updated in February 2020.

The study will follow ISO standards TC314 (Ageing committees) and 82304-2 (Health and wellness apps), as noted in the AAL Guidelines for Ethics, Data Privacy, and Security, as they are also relevant.

However, due to the openness that a participatory design approach brings to research projects, fixed regulations and ethics guidelines may not suffice for Anathema. Therefore, an in-action ethics [61] approach, whereby the entire consortium is called to define the project ethos, eliciting values that the project should stand for and that should guide decisions in case the answer to ethical dilemmas falls outside normative ethics principles, is being adopted.

#### Study Initiation

The pilot feasibility trials will be initiated in February 2023. The study duration will be approximately 12 months. A study initiation visit will be performed to present the research protocol, train the local study teams, and adjust the study procedures to site specificities. A training course targeting the study psychologists will be delivered before the onset of the recruitment period to train them on Anathema’s key components, structure, and delivery, as well as on the study procedures. This course will be delivered online by certified psychologists and sex therapists.

#### Recruitment

Recruitment procedures will follow a multicenter strategy, as different consortium partners have access to different study populations. Unie KBO in the Netherlands, and Instahelp and Sigmund Freud University in Austria will be responsible for recruiting older adults. In Portugal, IPO-Porto and Europacolon will be responsible for recruiting colorectal cancer survivors. Centro de Reabilitação Profissional de Gaia will be responsible for recruiting stroke survivors in Portugal. With the support of local study team members, potential study participants will be identified by reviewing patient files at each site or following advertisements of the studies in institutional newsletters, social networks, or newspapers.

#### Consent Process

All participants will provide informed consent before any study-specific assessments and procedures are performed. Regardless of the implemented recruitment strategy, a local study team member will perform the first contact to secure the participants’ permission to be approached by the researchers. Copies of the study information sheet and informed consent forms will be provided for analysis and discussion. Subjects self-referring to participate (eg, following the study’s advertisement) will be directly contacted by the study team to provide any required clarifications before giving their consent. After informed consent is obtained, screening and baseline assessment procedures may occur.

#### Screening and Baseline Assessments

Screening and baseline assessments will be conducted by certified psychologists or psychology students supervised by the study team. The abovementioned outcome measures will be used for this purpose (Table 3). Participants fulfilling the eligibility criteria for the study will then be further assessed via a diagnostic interview. The Mini-International Neuropsychiatric Interview (MINI) [52] will structure this process. Participants with severe mental disorders will be referred to psychology departments at study sites or community mental health services.

#### Enrollment and Randomization

Participants fulfilling all eligibility criteria will be registered and randomized. Simple randomization will be performed by an independent statistician, using a computerized program. Older adults in Austria, and colorectal cancer and stroke survivors in Portugal will be submitted to subsequent simple randomization so that they are randomly assigned to one of the study psychologists. Colorectal and stroke survivors will be guided by study psychologists from SexLab, and older adults in Austria will have access to blended support provided by clinical psychologists from Instahelp. Older adults in the Netherlands will not be submitted to this second randomization procedure, given that they will get access to the self-guided version of the program. Participants randomized to Arm A (experimental condition) will receive immediate access to Anathema. Participants assigned to the WLC condition will gain access to the program approximately 8 weeks after randomization and after being submitted to a second baseline assessment to confirm their eligibility for the study.

#### Study Intervention Delivery and Assessment

After randomization, participants will gain access to Anathema either via the co-developed app (older adults in the Netherlands, and colorectal cancer and stroke survivors in Portugal) or the Instahelp platform (older adults in Austria). At this stage, the 3 abovementioned service models will be tested for acceptability, feasibility, and preliminary efficacy.

The self-guided model will be tested with older adults in the Netherlands. Participants will gain access to the treatment modules every week, being prompted to complete the modules in approximately 1 week. If participants fail to complete the modules in the specified time frame, a reminder will be sent. Upon completion, participants will receive access to the following module. The preliminary efficacy of the intervention will be assessed at midtest (T2) and after the completion of the program at posttest (T3), using the measures listed above.

The guided model will be trialed by colorectal cancer and stroke survivors in Portugal. Participants will gain access to the intervention after a preliminary online or telephone appointment is conducted with their assigned study psychologist. This first e-appointment aims to discuss the program’s structure, report the baseline findings, and tailor the intervention according to participants’ needs and preferences. Once clinical psychologists and participants reach an agreement, the selected modules will be prescribed and made available weekly to the participants. Participants will be prompted to complete the modules in approximately 1 week. Within 24 hours of module completion, the study psychologists, based on the reported outcomes, will assess participants’ progress and determine whether it is appropriate or not for the participants to proceed to the next module. If so, participants will receive access to the following module. If not, study psychologists will instruct participants on what needs to be completed to advance to the next module. The preliminary efficacy of the intervention will be assessed at midtest (T2) and after the completion of the program at posttest (T3), using the measures listed above.

The blended model will be assessed by older adults in Austria. Participants will gain access to the intervention after a preliminary 60-minute videoconference appointment is conducted with their assigned study psychologist. This first appointment aims to discuss the program’s structure and tailor the intervention according to participants’ needs and preferences. Once clinical psychologists and participants reach an agreement, the selected modules will be prescribed and made available every week to the participants. Participants will be prompted to complete the modules weekly. Within 24 hours of module completion, the study psychologists, based on the reported outcomes, will assess participants’ progress and determine whether it is appropriate or not for the participants to proceed to the next module. If so, the participants will receive access to the subsequent module. If not, clinical psychologists will instruct participants on what needs to be completed to advance to the next module. Online videoconference appointments will be conducted at the onset of the program (session 1), at week 3/4 (session 2), and at the end of the program (session 3) to blend self-care work and asynchronous support with therapeutic videoconference synchronous support. During the videoconference appointments, study psychologists will assess participants’ progress, instruct participants on how to implement treatment strategies, and determine whether it is appropriate or not for participants to proceed with the program. Upon request, sessions are also possible for participants requiring additional support from clinical psychologists. Still, they are limited to 5 extra sessions, at a maximum of 1 per week and for approximately 40 to 50 minutes. If an extra videoconference session is requested, it will replace the scheduled asynchronous session to avoid redundancy. The preliminary efficacy of the intervention will be assessed at midtest (T2) and after the completion of the program at posttest (T3), using the measures listed above (Table 3).

#### Discontinuation of Participation

Participants may withdraw from the study at any time and for any reason, without any consequences, or be dropped from the study by the research team if a severe aggravation of baseline measures occurs. In this case, the participant will be referred to psychology services at study sites or specialized resources in the community. The main reason for withdrawal will be registered, and a final assessment will be performed whenever the participant agrees with it. Participants who are lost to follow-up may be replaced to ensure a balanced number of participants in each study arm.

#### Data Handling and Storage

All data to be collected within the scope of Anathema will be completed by participants via the Anathema app and through regular outcome assessments. Questionnaire data will be collected through LimeSurvey [62] and Microsoft Office Forms. These surveys will exclude personal information and IP storage, so that confidentiality and anonymity are ensured. Each participant will be assigned a random user, and this will be used by researchers to identify the participants and their survey responses during the study. Feasibility and trial-related data will be monitored via TrialMonitor [63], a software tool that scaffolds the creation of personalized web dashboards for monitoring participants in technology-enabled field trials. Data will be kept confidential and will be stored in compliance with physical infrastructure and software requirements imposed by the European Union (EU), namely the EU Regulation 2016/679 of The European Parliament and of the Council of April 27, 2016, on the protection of natural persons with regard to the processing of personal data and on the free movement of such data. The questionnaire responses and data from the application will be stored in a secure database that is not accessible from the outside world without proper authorization. The data will be protected by multiple layers of security to ensure that it remains confidential and can only be accessed by authorized personnel. The database will be regularly backed up to ensure that the data are not lost in case of any unforeseen events. Additionally, strict protocols will be in place to track and monitor any access to the data, to ensure that the data are only being used for authorized purposes. All communication related to Anathema will take place within the developed platform, so no confidential information is sent unencrypted and via third-party programs.

Regarding physical infrastructure, data will be stored in servers that are physically located on the premises of Associação Fraunhofer Portugal Research in Porto, Portugal. These servers are situated in a locked server room that is equipped with a key and card-keypad access control system, and is accessible to only authorized personnel. A dedicated division of IT technicians, working at Associação Fraunhofer Portugal Research, is responsible for the maintenance of the hardware equipment (servers and network), ensuring that all equipment is constantly running and is redundant (various hardware backups ensure continuity if one system fails). The software on the servers is updated constantly. Additionally, full backups of the system are performed on a daily basis and stored at a different location than the live servers. These backups are encrypted. The server operating system is Linux-based, and the operating system installation is dedicated only to the platform services. Encrypted (https/Transport Layer Security) protocols will be used in all data communication between the servers and the users, as well as in peer-to-peer audio/video chat. The key to produce the code that allows the indirect identification of the participants will be deleted 5 years after the end of the study, as mandated by Deliberação n. 1704/2015 (Deliberação da Comissão Nacional de Proteção de Dados Aplicável aos tratamentos de dados pessoais efectuados no âmbito de Investigação Clínica).

Instahelp uses 3 different security mechanisms to protect data from external access: (1) encryption of data before transmission (end-to-end encryption), (2) secure data transmission (Transport Layer Security), and (3) 2-factor authentication. The widely used and recognized LibSodium cryptography library is used to encrypt the data. Each individual chat message is encrypted with a symmetric space key based on the Salsa20 (64-bit) method. In addition, each message is checked for tampering after transmission using message authentication via Poly 1305MAC. The hosting of the web server (including the implementation on the server side) and the database are provided by the IT service provider 1&1 in Frankfurt (Germany).

### Analysis

Descriptive statistics (ie, means, percentages, frequencies, etc) will be used to characterize the study sample and report on acceptability, usability, and feasibility quantitative measures. Acceptability and feasibility assessments will also be based on the qualitative data collected during the postintervention interviews. Qualitative data will be thematically analyzed [64].

To evaluate Anathema’s preliminary efficacy, we will first perform independent sample *t* tests and chi-square analysis to assess potential baseline differences between participants randomized to the experimental and control arms. Second, we will calculate the differences between the experimental group and the control condition for secondary outcomes. Data analysis will be performed according to the intention-to-treat principle and resorting to a mixed-models approach [65]. Effect sizes (Cohen *d*) [66] and the 95% CI will be calculated to measure the treatment magnitude for continuous outcomes. Finally, clinical significance will be assessed using the Jacobson & Truax method [67]. All statistical analyses will be conducted in IBM SPSS for Windows (IBM Corp).

## Results

The European Commission funds the project Anathema through the AAL Programme (reference: AAL-2020-7-133-CP) from April 2021 to December 2023. Recruitment for the pilot RCTs started on January 2023 in Portugal, Austria, and the Netherlands, and is currently ongoing. As of May 2023, we randomized 49 participants in the trials. We expect to complete the RCTs in September 2023. The results on the acceptability, feasibility, and preliminary efficacy of Anathema are expected in the second semester of 2023.

Considering that the development of Anathema involved a participatory design approach [40] and included primary, secondary, and tertiary end users, we expect it to be highly accepted by older adults, colorectal cancer survivors, stroke survivors, mental health professionals, and sexual health experts. We also expect it to prove feasible to scale up to parent RCTs and to be potentially efficacious in improving the relationship and sexual satisfaction, sexual pleasure, and HRQoL of older adults, colorectal cancer survivors, and stroke survivors compared to TAU in a WLC condition. Study results will be published in open-access venues according to COREQ (Consolidated Criteria for Reporting Qualitative Research) [68] and CONSORT EHEALTH (Consolidated Standards of Reporting Trials of Electronic and Mobile Health Applications and Online Telehealth) [69] guidelines.

## Discussion

This study is designed to assess the acceptability, usability, feasibility, and preliminary efficacy of Anathema in decreasing sexual distress and improving sexual function, relationship and sexual satisfaction, sexual pleasure, and HRQoL in older adults, colorectal cancer survivors, and stroke survivors when compared to TAU in a WLC condition. We expect this feasibility study to prove that the adopted methodology is sound, feasible, and coherent with participants’ expectations and needs. We also expect Anathema to be potentially efficacious in improving the relationship and sexual satisfaction, sexual pleasure, and HRQoL of older adults, colorectal cancer survivors, and stroke survivors compared to TAU in a WLC condition.

To our knowledge, this is the first study evaluating the acceptability, feasibility, and preliminary efficacy of a smartphone-delivered sexual health promotion program targeting older adults that also caters to the unmet specific sexual health needs of colorectal cancer and stroke survivors, and is an important contribution to this knowledge field. Furthermore, the obtained results will add to the evidence on the acceptability, feasibility, and effectiveness of internet-delivered sex therapy in cancer [25-27] and chronic disease patients [24]. The results will also inform Anathema’s refinement and the methodology used in future parent RCTs testing the effectiveness of the sexual health promotion program in the 3 target populations.

If proven acceptable, feasible, and preliminarily effective, Anathema will be made available to participants, and health care and research organizations to be used free of charge in health care and future research. Anathema is expected to be an important self-care tool to be used autonomously and with the support of sexual health experts, contributing to close the sexual health care gap experienced by older adults, colorectal cancer survivors, and stroke survivors.

The strengths of this study include the inclusive, participatory, and multicountry design approach used in Anathema’s development. This approach will ensure Anathema is culturally sensitive in the Netherlands, Austria, and Portugal, facilitating the program’s acceptance and use in these countries. The fact that the program is inclusive and made available in English also enables the opportunity of conducting future research targeting other populations and countries. Regarding the study design, the multicentric feasibility RCT design to be used in the pilot phase is of utmost relevance, as it will provide information on Anathema’s feasibility and preliminary efficacy, and increase the chances of the generalizability of the results. The assessment of 3 different service delivery models for Anathema in comparison with TAU is also an advantage, as it will provide data on their relevance and preliminary impact in the target populations, informing the design of future sexual health care services. Finally, the combination of the RCT design with the qualitative insights emerging from the posttest interviews will warrant deeper exploration of Anathema’s acceptance and future use, facilitating its future implementation.

The potential limitations include challenges in the recruitment and retention of participants. The limited research conducted to assess the acceptability, feasibility, and efficacy of smartphone-delivered sexual health promotion programs in older adults, colorectal cancer survivors, and stroke survivors makes it difficult to estimate recruitment, retention, and adherence rates for Anathema. To overcome this limitation, a multicountry participatory design approach was used to design Anathema, and diversified recruitment strategies were defined (eg, local recruitment by clinical teams at each site, and advertisements of the studies in institutional newsletters, social networks, or newspapers).

## Appendix

Multimedia Appendix 1 Peer review report from the Active and Assisted Living Programme - AGE Platform Europe - European Commission (Brussels, Belgium).

## Abbreviations

AAL: Active and Assisted Living
HRQoL: health-related quality of life
IPO-Porto: Instituto Português de Oncologia do Porto Francisco Gentil
RCT: randomized controlled trial
TAU: treatment as usual
WLC: waiting-list control

## Data Availability

The data sets generated during and/or analyzed during this study are available from the corresponding author on reasonable request.
