# Supplementary material for: A Smartphone-Delivered Program (Anathema) to Promote the Sexual Health of Older Adults, Colorectal Cancer Survivors, and Stroke Survivors: Protocol for a Feasibility Pilot Randomized Controlled Trial
Source: JMIR Res Protoc. 2023 Jun 27;12:e46734. doi: 10.2196/46734 (PMC10337355; doi:10.2196/46734)
Supplement: Multimedia Appendix 1 [file resprot_v12i1e46734_app1.pdf]

The Active Assisted Living Programme

Evaluation Summary Report (ESR) -

Evaluation-call-2020

PROPOSAL: Technology for ageless sexual health

aal-2020-7-133-CP

1. RELEVANCE AND SCOPE — 4 / PASSED

Anathema focuses on the Health & Care TAALxonomy and Telecare and Telehealth, in particular digitalizing sexual health interventions for increased adherence and scalability with a focus on 3 primary user groups (older adults, colorectal cancer and stroke survivors). The proposed solution seeks to address issues relating to sexual health, which is central for wellbeing and quality of life, but not adequately substantiated because interventions target other user groups (e.g. adolescents) or because people do not seek sexual health support. The project outputs are suitable both for consumer and institutionalized markets.
Anathema positions itself as the first smartphone-delivered sexual health promotion intervention for older adults and people with chronic diseases, available in 4 languages, therefore demonstrating new use of technologies to support older adults.
The example scenario is engaging and clearly presents the modular functionality of the proposed solution and the value proposition for both primary and secondary end-users. The idea is to develop a solution which adapts traditional face-to-face sexual health promotion interventions to be delivered through mobile devices, tailoring them for older adults, including those with chronic diseases (and their partners), and to make them highly usable. The proposal responds to a growing demand and presents the potential impact for health practitioners in terms of decreasing intervention drop-out, and boundless geographically coverage.
The tackled challenges are of interest for a large set of end-users (primary, secondary and tertiary), thus confirming a good market potential. In particular, three primary user groups will be involved in the project. Moreover, psychologists, sexologists (secondary end users) and care organizations (tertiary end users) have been considered.
Regarding the technical development, the proposal is based on an existing commercial platform (INST, TRL9) but recognizes the critical need to evidence effectiveness. The Google H.E.A.R.T. framework will be used to evaluate Happiness, Engagement, Adoption, Retention and Task Success through a remote monitoring tool (currently at TRL7). Also, the proposal will build on an existing psychology/sexology service/platform to include specific modular support for the primary user groups and can then therefore be scaled to reach other user groups. The proposal presents a very clear summary of its fit to the call. It also proposal demonstrates the potentiality of the proposed ICT solution to address the defined challenges. In particular, the proposed approach will use Anathema smartphone apps, a desktop app for psychologists to tailor interventions, a repository for the intervention modules and a tool for remote monitoring of the technology to track Anathema use during the pilot studies. The proposal is convincing in its confidence to test redesigned service models with primary, secondary and tertiary end-users for reliability, security, financial viability and ease of integration. Commitment to the methodology is evidenced in the form of letters of support from stakeholders and Advisory Board participants and partners providing access to relevant end-users. The European dimension of the consortium and the Advisory Board is justified as critical, given the cultural distinctions related to the sensitive topic.
The proposal demonstrates a possible positive impact of being sexually active on Health, Wellbeing and Quality of Life, and how this is threatened by Long Term Condition’s and that the challenge is underserved. The target 3 user groups are fully justified as older adult sub-groups that are potentially most affected, for example, stroke for its debilitating effects and ability to use technology. The consortium declare there are no known specialist apps that focus on sexual health for older adults / chronic diseases, but benchmark against the closest competitor products. An overview is provided of the nearest state of the art research, its limitations, and how Anathema can build and improve upon these baseline interventions.
Moreover, a well elaborated benchmark is presented in Table 2 (section 3.2.1).
This being said, the novel elements provided are significant to create a unique selling point (USP) in comparison to state-of-the-art solutions.
The consortium leverages prior research and knowledge into their design principles, such as the use of humor to break down taboo’s, and understanding the value in serious games. An inclusive design approach will address a wide range of user needs e.g. voice interaction instead of text. The KPI’s are clear, relevant and measurable. The key performance indicators have a good mix of subjective and objective criteria with data being captured in a systematic manner that will aid in product development and refinement. These KPI's will also help define success for the consortia in the process.

2. IMPLEMENTATION - QUALITY OF PROPOSED SOLUTION AND WORKPLAN — 4 / PASSED

The consortium seems well versed in the challenges of a participatory co-design approach and the need to manage shifting priorities. Equal importance is put on primary, secondary and tertiary end-users, which is a strength. The approach is very clearly

detailed with defined numbers, extensive inclusion/exclusion criteria and quantified and measurable co-design, testing and evaluation activities across primary, secondary, and tertiary end-users. Numbers are suitable to deliver a scientifically robust evaluation, field trials being setup in three countries with three user groups in order to be able to demonstrate various use cases.

Each user-group will also input into the various end-products relevant to them e.g. standalone app, intervention modules for secondary end-users, and institutional packs for tertiary end-users.

Extensive end user engagement through the participatory design approach has been identified during the designing and testing of the application and the embedded modules. The evidence based solution being proposed will also help to engage the market. The downside of this approach is potential lack of focus with a number of disparate groups across effectively multiple ecosystems being engaged in the development of the product. This could lead to complexity in designing the Minimum Viable Product (MVP) whereas speed to market through perhaps focusing purely on seniors in the first instance, may allow for a product to be launched and then refined through real time user experiences, thereafter delivering v2.0 and v 3.0 as the user sets are broadened.

Ethics is handled proficiently and with attention to continuous end-user involvement with specific consent forms for each activity. An In-Action Ethics approach will be adopted and data will be robustly managed via the Ethics and Data Protection Manager. The reliability/security/privacy related functionalities have been well addressed in the proposal. In particular, the proposal clearly states that Anathema will be compliant with GDPR in its legal formulation in each of the participating countries.

Necessary attention with users is given to defining the value proposition and understanding purchasing decisions to quantify a Return On Investment. Living environments to perform testing activities with end-users have been sufficiently specified in the proposal. The Advisory Board provides access to nursing homes, but the proposal does not directly address the barriers and taboo's that still exist in care and nursing homes, where residents' sexual needs are traditional not met with a lack of structure in place to support the need for resident's intimacy.

It is positive to see that an MVP is aimed for as early in the project as possible to allow for continued iterative improvements thereafter. The technical components and the envisaged final Technology Readiness Level (TRL) is presented with user interfaces for both Android and iOS. Indeed, a very good description of the technical activities to be developed within the project has been elaborated, with particular regards to the intervention modules, the module repository, the smartphone application, the web application, and the remote monitoring tool. Satisfactory attention is given to data privacy and security. Motivations leading to estimate TRL7 for the Anathema platform at the end of the project and TRL8 for the integration in INST's platform are convincingly elaborated in the proposal.

The proposal appropriately describes how stakeholders will be engaged in project activities with particular regards to development and commercialization, as also demonstrated by the man effort and task breakdown through different WP's. In particular, for product distribution, INST will use its existing customer base and techniques for client acquisition, while SPR will exploit existing contacts of end-user organizations in The Netherlands.

It is worth noticing that after the project, the technology will remain available to R&D and end-user partners for future development and exploitation. Also, the approach towards interoperability and the relevant standards is clearly depicted in the project. In particular, standard devices and communication protocols will be used to assure the integration of the solution proposed in standard platforms. Open standards will be used to create an interoperable solution and ensure easy integration and operation via smartphones and psychologists' desktops.

3. IMPLEMENTATION - QUALITY OF CONSORTIUM AND PROJECT MANAGEMENT — 5 / PASSED

The consortium involves South, Central and Northern Europe to span different cultures with varied beliefs regarding sexuality. There is equal effort across business, research and end-user partners. Also, there is a confident approach to the user involvement and field trials given the consortium's expertise. The proposal clearly demonstrates that the consortium have the necessary expertise to reach the project goals. Indeed, the complementarity between the consortium members justifies fully undertaking the project using European partners rather than using national partners only. It must be underlined that the Anathema project has drawn the attention of organizations who have interest in the project results and have agreed to take part without any funding, as demonstrated by "intent letters." Furthermore, these letters of intent demonstrate a good level of interest from a patient association, oncology hospital, and psychology research group who will contribute non-funded to the project with a view to integrating the outcomes in their platform. There is also early interest from a private clinic in Spain, and Advisory Board members are secured extending the geographical coverage of the project.

The consortium's success is likely strengthened by the use of INST's mental health platform as its foundation, whilst overall, the partners expertise suitably spans design research, sexology, digital health, psychology, oncology, gerontology and AAL markets. Each partner and non-funded contributor are clearly presented, highlighting the expertise they bring to the project. The way in which the competence of the project partners is mapped to the tasks to be performed is convincing. This assessment is based on the profile of partners which have been clearly demonstrated in the proposal, concerning both technical and marketing skills.

The partner-conflict-resolution strategy is sufficiently addressed in the proposal (see 2.6).

The consortium has chosen to run a 24 months project reiterating the rapid sprints previously mentioned. The project plan supports that this is feasible with all partners having some level of responsibility in all work packages. The only concern is the multiple user groups which adds a level of complexity that may result in a MVP not being defined in a timely manner as different user groups drive different changes to the core platform. The work packages are sound and relevant to the project objectives. Dependencies are presented and deliverables are thoroughly detailed and made public where appropriate. The funding requested from AAL, including breakdown of costs and major budget requests are well set out and is in line with the expected results. This assessment is based on the amount of activities planned in the proposal and resources required for the solution development. Moreover, the description of how resources will be deployed to the various work activities and through the project timescale is well set out and convincing and well described in terms of PM's (see Tables on pages 21-22).

There is confidence in the recruitment strategies given potentially even more challenges for a taboo subject. 4 of the partners and 2 non-funded organizations have ready cohorts across the 3 primary end-user groups. To further support the recruitment strategy, minimal sub-contracting is justified as crucial to secure patient recruitment and psychologist support where it isn't available.
The risk analysis includes the possibility of the project being carried out remotely to respond to Covid-19. A sound risk analysis and suitable mitigation strategies are presented spanning technical, scientific, managerial, and commercial risks. The consortium could spend more time prior to starting the project confirming the risk profile of the complex multi-user, multi-jurisdictional project when delivering the app platform. That said, this is mitigated by using a proven, on market technology as the baseline tech.
The consortium demonstrates their AAL project management experience and shows an appreciation of what standard of reporting is expected. This runs through the risk mitigation strategies and the deliverables, in which they recognize WP3 prototype deliverables will benefit from written proof of development. This project management expertise continues into the proposed management structure, with defined reporting and clear decision making processes. The management organization of the project is clearly elaborated. Specifically, a lean management structure has been chosen, which includes decision making and operational bodies. Given the project nature, ethics and data are to be robustly managed by an Ethics and Data Protection Manager adhering to all national and European law.

4. IMPACT - POTENTIAL IMPACT OF PROPOSED SOLUTION ON QUALITY OF LIFE — 4 / PASSED

The development of an innovative, evidence based sexual health program that can be delivered digitally could be a game changer for older persons quality of sexual life. The project may allow for wide engagement across the market segment, allowing what had previously been a difficult discussion to be explored in a more private yet effective way. Improvement in both physical and mental health for older persons could be generated with the use of Anathema. Significant evidence and references support the fact that a positive sex life results in good psychological and physical health which in turn positively impacts on quality of life, and the reverse can be said. This area often goes untreated given the taboo's, and so the proposed discrete and ubiquitous solution aims to overcome said treatment barriers. The proposed solution represents significant improvement in the quality of life of the targeted end user group(s). For primary end-users, the solution aims to enhance sexual-health related issues. For secondary end users, such as psychologists/sexologists, Anathema will become an evidence-based and convenient tool to deliver sexual health interventions with the potential to improve healthcare outcomes and increase business size. Moreover, users living and working in nursing homes will also exploit advantages coming from the solution proposed.
The potential benefits to secondary end-user practitioners are plentiful, offering remote access to their patients removes physical boundaries with the potential to expand their business even in pandemic times. The evidence-based tool could also educate and create a shift in nursing home's culture to recognize the important of intimacy on resident's quality of life. The Care Quality Commission in the UK has already recognized that care homes must address resident's intimacy needs or risk failing inspections.
The proposal convincingly demonstrates that the proposed solution will positively impact on current service models. In particular, two main service models in delivery of sexual health interventions have been addressed in the proposal, face-to-face and remote. Since no smartphone-mediated sexual health interventions focusing specifically on issues for older adults and people with chronic diseases has been identified in the market, there is comparative advantage.
The proposal demonstrates that commercial partners have a suitable understanding of the market opportunities, established routes to market, and a cost/benefit analysis (Sec. 3.2.1).
Also, the proposal addresses its alignment with service models enabling services to move online and how it can be used in self-guided, guided (with a psychologist) or blended modes. For scalability, the self-guided mode can be independently commercialized and it does not qualify as a medical device and therefore does not require certification. The project will also test using the application as a training tool for non-specialist psychologists. Costs for primary end-users are not anticipating to be different to current counselling services.
Ethical aspects have been elaborated in the proposal, which indicate a good understanding of the real-life challenges experienced by older adults and people with chronic diseases facing sexual-health related issues. Moreover, issues related to personal data management and issues occurring during pilot trials with end-users have been considered (see section 3.1.4). The proposal is convincing when it comes to managing end-user studies and substantial awareness is shown regarding ethical concerns and the handling of any negative impact that may be experienced by trial participants whilst also understanding that positive impact will be slow to be declared. To this effect the project aims to deliver awareness campaigns. The management of departure/loss of end-user participants is clearly developed. A duty of care is considered for any participant drop-outs with a referral to the relevant psychology department and a final assessment. Strategies have been put in place to reduce undesired side effects, such as the use of users' own smartphones during trials to avoid the need to carry two smartphone or adapt to the use of a new device. By using the participants own smart phone the proposal provides an exit strategy as a self-guided version can continue after the project.

5. IMPACT- POTENTIAL IMPACT OF PROPOSED SOLUTION ON MARKET DEVELOPMENT — 4 / PASSED

Overall, the consortium recognizes that given the subject nature the commercialization risk is potentially high, but the consortium is well placed to mitigate this with an experienced lead commercial partner with a network of 120 psychologists that can quickly add Anathema to its existing business model. Anathema will be built on an existing commercial platform as well as non-existing therapy services in clinical and third sector settings. As such, the probability of failure on technical development and market engagement should be significantly reduced.
A comprehensive competitor analysis is presented confirming that the nearest state of the art does not address the needs of older adults or those with chronic conditions specifically on sexual health. The analysis at least provides an indication of similar apps, their main features and price. Competitors appear to target certified therapists whose clients would be eligible for insurance rebates

- ultimately the institutional markets of public health authorities or insurers. The existing business model may need to be adapted to insure that revenue is captured from those willing to pay. It is great to see a benchmarking study against competitors when determining the business model, and approach to generating revenue. This provides Anathema the opportunity to better understand market positioning and how to deliver a unique selling proposition. Commercialization partners (mainly INST and SPR) will exploit their already established market network to launch solutions developed through the project. The main targets to be addressed are older adult and patient associations, user referrals, and professional societies. Such targets will be activated already during the project, by feeding surveys to secondary and tertiary users. In particular, Anathema will improve the two business partners’ business models, to be exploited in 4 countries: 1) via the app stores as a stand-alone app (NL), and 2) as an immediate add-on to INST's existing online psychotherapy platform (AT, CH, DE, NL). Hence, two business models will be developed.
Early Participatory Design will shed indications as to the level of primary end-user openness and therefore will inform which cultures are predominantly more likely to adopt. A convincing attempt is made to demonstrate the need and likely demand based on user data from INST and to quantify the addressable market for each of the 3 target user groups. One route to market is to white label via specialized clinics which are identified and listed in the proposal. A lean Business Model Canvas is presented and pricing strategies in line with traditional face to face counselling are explored. However, the planned timescale to reach the market is not fully identified in the proposal. The dissemination plan is appropriately developed and target groups have been conveniently identified. The plan foreseen also has actions aimed to assess the effectiveness of outreach and scientific dissemination activities. The dissemination plan is convincing and is well thought. The coordinator and INST have a sound understanding of how to manage IPR fairly. A specific IPR agreement will go beyond that set out in the collaboration agreement. Indeed, the consortium will use DESCA2020 model. D5.3 will also cover the IPR protection within the project.

**6. REMARKS**	**NOT RATED**

TOTAL SCORE: 21
